# A Metagenomics Study on Hirschsprung's Disease Associated Enterocolitis: Biodiversity and Gut Microbial Homeostasis Depend on Resection Length and Patient's Clinical History

**DOI:** 10.3389/fped.2019.00326

**Published:** 2019-08-09

**Authors:** Alessio Pini Prato, Casey Bartow-McKenney, Kelly Hudspeth, Manuela Mosconi, Valentina Rossi, Stefano Avanzini, Maria G. Faticato, Isabella Ceccherini, Francesca Lantieri, Girolamo Mattioli, Denise Larson, William Pavan, Carlotta De Filippo, Monica Di Paola, Domenico Mavilio, Duccio Cavalieri

**Affiliations:** ^1^Division of Pediatric Surgery, AON SS Antonio e Biagio e Cesare Arrigo, Alessandria, Italy; ^2^Division of Pediatric Surgery, Giannina Gaslini Institute, Genoa, Italy; ^3^Department of Dermatology and Microbiology, University of Pennsylvania, Philadelphia, PA, United States; ^4^Unit of Clinical and Experimental Immunology, Humanitas Clinical and Research Center, Milan, Italy; ^5^Department of Medical Biotechnologies and Translational Medicine (BioMeTra), University of Milan, Milan, Italy; ^6^Department of Neuroscience, Rehabilitation, Ophthalmology, Genetics and Maternal and Child Science (DINOGMI), University of Genoa, Genoa, Italy; ^7^UOC Medical Genetics, Giannina Gaslini Institute, Genoa, Italy; ^8^Biostatistics Section, Department of Health Science, University of Genoa, Genoa, Italy; ^9^Genomics, Development and Disease Section, National Human Genome Research Institute (NHGRI), National Institutes of Health (NIH), Bethesda, MD, United States; ^10^Institute of Agriculture Biology and Biotechnology, National Research Council, Pisa, Italy; ^11^Department, of Biology, University of Florence, Firenze, Italy

**Keywords:** metagenomics, enterocolitis, Hirschsprung, *RET* gene, aganglionosis

## Abstract

**Objectives:** Since 2010, several researches demonstrated that microbiota dynamics correlate and can even predispose to Hirschsprung (HSCR) associated enterocolitis (HAEC). This study aims at assessing the structure of the microbiota of HSCR patients in relation to extent of aganglionosis and HAEC status.

**Methods:** All consecutive HSCR patients admitted to Gaslini Institute (Genova, Italy) between May 2012 and November 2014 were enrolled. Institutional review board (IRB) approval was obtained. Stools were sampled and 16S rDNA V3-V4 regions were sequenced using the Illumina-MiSeq. Taxonomy assignments were performed using QIIME RDP. Alpha diversity indexes were analyzed by Shannon and Simpson Indexes, and Phylogenetic Diversity.

**Results:** We enrolled 20 patients. Male to female ratio was 4:1. Six patients suffered from Total Colonic Aganglionosis (TCSA). Considering sample site (i.e., extent of aganglionosis), we confirmed the known relationship between sample site and both biodiversity and composition of intestinal microbiota. Patients with TCSA showed lower biodiversity and increased Proteobacteria/Bacteroidetes relative abundance ratio. When addressing biodiversity, composition and dynamics of TCSA patients we could not find any significant relationship with regard to HAEC occurrences.

**Conclusions:** The composition of HAEC predisposing microbiota is specific to each patient. We could confirm that total colon resections can change the composition of intestinal microbiota and to dramatically reduce microbial diversity. The subsequent reduction of system robustness could expose TCSA patients to environmental microbes that might not be part of the normal microbiota. Future long-term studies should investigate both patients and their family environment, as well as their disease history.

## Introduction

Enterocolitis (HAEC) is an extremely serious, life-threatening complication that can occur in children with Hirschsprung disease (HSCR) pre- and even post-operatively. Despite a number of studies, the causative agents of HAEC are still elusive. Standard culturomics technologies did not lead to the discovery of microbial pathogens causing HAEC. Novel culture independent approaches based on DNA sequencing of target genes or of the whole bacterial DNA content hold the promise to discover the causative agents and the etiology of HAEC. Since 2006 (1) metagenomics in children has been used to address the role of intestinal microbiota in the etiology of a number of diseases (1–11). Only a minority of papers addressed HSCR and HAEC (6, 12–14). In 2010, our group carried out a pilot genomic study in a single patient with HSCR and demonstrated that HAEC can correlate to changes in gut bacterial dynamics (12). Ward and co-workers later on reported a sustained abnormal microbiota in an animal model of HSCR (13). Yan in 2014 (6) and Frykman in 2015 (14) investigated HSCR patients with or without HAEC. Both authors did not find any significant difference apart from the latter who reported an altered *Candida* community in those with HAEC (14). Of note, Li et al. in 2016 reported a significantly different microbiome in HSCR patients with HAEC (15). Proteobacteria were significantly more represented in patients with HAEC whereas Bacteroidetes were significantly more represented in patients without. Also HSCR patients had a relatively distinct, more stable community than the HAEC and HAEC-R patients (previously settled HAEC episode), suggesting that enterocolitis may either be caused by or result in a disruption of the patient's uniquely adapted intestinal flora. The intestinal microbiota associated with enterocolitis may persist following symptom resolution and can be implicated in symptom recurrence. In another recent study on a mouse model of HSCR, the metagenomics analysis of *Ednrb*^−/−^ and wild type mice showed that mutants had a distinct microbiota with respect to wild type (WT) and that the HAEC group had lower alpha diversity by Chao1 index compared with WT. Also the animals with HAEC had increased proportion of *Akkermansia* genus and reduced Bacteroidetes phylum compared with the NO HAEC and WT groups, suggesting *Akkermansia* may contribute to development of enterocolitis while Bacteroidetes may be protective (16). Finally, another study on Finnish patients showed how those with HD and HAEC had a significantly altered intestinal microbiome compared to healthy individuals, characterized by a lack of richness and pathologic expansions of taxa, particularly Enterobacteria and Bacilli (17). These initial studies did not lead to a predictive profile for HAEC (18, 19). Nonetheless, no mention was done either regarding extent of aganglionosis or diversity measures (15). Here we report results of a metagenomics study on fecal microbiota performed on HSCR patients, addressing limitations, drawbacks and potential benefits of such approach, delineating future perspectives in this field of research.

## Methods

### Patients

All pediatric patients with HSCR consecutively admitted to Giannina Gaslini Institute (Genova, Italy) between May 2012 and November 2014 were eligible for this study. Institutional Ethical committee approval was obtained by the Review Board of Giannina Gaslini Institute on November 2009 as part of a wider research project on HSCR. A specific informed consent was signed by all participant families. Inclusion criteria were: (1) diagnosis of HSCR based on histochemical assessment of adequate rectal suction biopsies, as previously reported (20); (2) exhaustive data regarding extent of aganglionosis (adequate intraoperative histology) and regarding personal history with specific regard to previous bouts of HAEC; (3) stool sampling available both from preoperative and postoperative periods. Exclusion criteria were (1) refusal of signing the informed consent; (2) failure to pass internal quality control; (3) inadequate sampling or storage.

### Definitions

HSCR, Hirschsprung's disease; RSA, HSCR with aganglionosis extended up to the colonic splenic flexure (i.e., Rectosigmoid Aganglionosis); L-HSCR, Long HSCR with aganglionosis extended beyond the splenic flexure up to ascending colon; TCSA, HSCR with aganglionosis extended to the whole colon (i.e., Total Colonic Aganglionosis); HAEC, Enterocolitis diagnosed according to the combination of Pastor criteria (21) (to confirm the diagnosis) and Elhalaby criteria (22) (to grade HAEC severity).

### Stools Collection, Storage, and Delivery

Spontaneous stools were collected and stored frozen (−20°C up to 48 h and −80°C afterwards) until shipment to the reference center for processing of fecal samples (Bethesda, Maryland, USA). Stools from Total Colonic Aganglionosis (TCSA) patients belonged to stoma bags preoperatively and from direct bowel movements postoperatively (in both cases from the ileum) whereas stools from Rectosigmoid Aganglionosis (RSA) or long (L)-HSCR belonged from bowel nursing (enema or rectal tube) preoperatively and from direct bowel movements postoperatively. Time-points for stool sampling were: (1) before surgery (Timepoint 1, preoperative hospital stay), (2) intraoperative (timepoint 2), and (3) postoperatively 7 to 10 after pull-through (timepoint 3). Only timepoints 1 and 3 (preoperative and postoperative, respectively) were assessed in this study.

### Clinical Features and Molecular Genetics

All patients included in this study underwent a thorough phenotype assessment as previously published (23). Demographic data, phenotype results, HAEC status, surgical details, possible complications and long-term outcome were recorded and stored in a digital database according to data protection Act. Furthermore, all patients underwent sequencing of the *RET* gene coding portion (21 exons flanked by at least 20 bp of intronic sequences) as part of the multidisciplinary diagnostic algorithm, as previously published (24). In order to identify mutations that are potentially involved in HSCR, the results of molecular genetics, consisting of (1) putatively pathogenic *RET* mutations; (2) common variants among which those known to represent HSCR risk modulating factors (25), were recorded in the same database as above.

### 16S rDNA Sequencing and Processing

Amplification and sequencing of the 16S rDNA hypervariable V4 region was performed as previously described using the Illumina MiSeq platform with 150 bp paired end reads and V2 chemistry (26). Sequences were demultiplexed with FLEXBAR (27) and assembled using PEAR (28). Sequences were then quality filtered and restricted to length of 245–255 nt, which retained 9.77 million reads of the filtered 9.78 million reads. The sequences were then analyzed using scripts within the QIIME package (29). Sequences were first clustered into *de novo* operational taxonomic units (OTUs) defined by 97% sequence identity using UCLUST (30). A representative set of sequences for the OTUs was selected using the QIIME “most abundant” selection method. Taxonomy assignments for the representative sequences were performed using the QIIME RDP wrapper with an 80% cut-off for bootstrap confidence in assignment (31). Unclassified sequences and reads classified as Cyanobacteria were removed. The representative sequences were then aligned using PyNAST with a minimum length of alignment cutoff of 150 nucleotides and a minimum percent identity cutoff value of 75% (32). Alignments were performed against the Greengenes 13_8 taxonomy core-set alignment sequences (33). Chimeras were removed from the aligned reads using ChimeraSlayer (34). ChimeraSlayer identified 21,593 OTUs (4.0%) out of 537,481 total OTUs as chimeric. OTUs that possessed <2 sequences and did not occur in more than one sample were removed. Taxonomy summaries were further filtered to only include OTUs that make up at least 0.5% of the total sequences. Rarefaction of the samples was performed on all samples at a depth of 7070 sequences to ensure homogeneity in sample size for downstream analysis. Phylogenetic tree construction of the aligned sequences was performed using FastTree (35). The phylogenetic tree was used for calculation of UniFrac (weighted and unweighted) beta diversity distance matrices (36).

### Statistical Analyses

The results were compared according to length of aganglionosis, timepoints, and HAEC status (HAEC episodes either experienced during the study period or before enrollment but reported in personal history (pre-HEAC). The single patient with L-HSCR could not be categorized as classic or ultralong HSCR and was excluded from this statistical analysis in order to avoid misinterpretation (Table 1). Diversity indexes as well as phylogenetic analysis were compared in patients with RSA and TCSA regardless of timepoints. TCSA patients that represented the core of our study underwent a further analysis based on HAEC status and timepoints of stool sampling. The R ggplot2 package was used for plotting (37). Alpha diversity index analyses of the samples were conducted using QIIME and the constructed phylogenetic tree to calculate the Shannon index (38), Simpson Index (39), and Phylogenetic Diversity (PD) (40). Pairwise comparison with Wilcoxon rank sum test was used to address Alpha diversity measures. Differences in categorical variables were addressed with chi-square or Fisher exact test, when appropriate. All tests were 2-tailed. A *p*-value lower than 0.05 was considered as statistically significant.

**Table 1:** Patients with TCSA in our series.

	**N. of pts**	**M:F ratio**	***RET* mutations (%)**	**Associated anomalies**	**HAEC**
RSA	13	5.5:1	3 (23%)	4 (31%)	1 (8%)
L-HSCR^*^	1	n.a.	0 (n.a.)	0 (n.a.)	1 (n.a.)
TCSA	6	2:1	4 (67%)	3 (50%)	3 (50%)
**DETAILS ON PATIENTS WITH TCSA (TIME POINTS COMPARED FOR DYNAMIC CHANGES)**					
**ID of TCSA**	**Gender**	**Age**	**Detailed** ***RET*** **mutation**	**Associated anomalies**	**HAEC**
A	M	15 months	None	CAKUT	Post severe
C	M	16 months	None	None	Pre severe
K	M	9 months	c.833C>A (p.T278N)	CAKUT	Post severe
L	M	20 months	c.820_820delG (p.A274Rfs^*^38) c.2075_2076delinsAA (p.A692E)	None	None
N	F	26 months	c.2772_2773insT (D925^*^)	GUT + EYE	None
Q	F	14 months	c.2829_2830insGGAG (p.I944Gfs^*^16)	None	None

*The prevalence of associated anomalies is in line to what previously published by our group (20). The table summarizes data regarding RET mutations, Associated Anomalies and HAEC status of the whole series of patients who underwent metagenomics, focusing on patients with TCSA who underwent a deeper assessment. None of the patients received antibiotics close to preoperative timepoint sampling. All TCSA patients received antibiotic prophylaxis for the first 3 days postoperatively (4 to 7 days before postoperative timepoint sampling)*.

*N, number; pts, Patients; n.a, not assessed; HAEC, Hirschsprung's associated enterocolitis; RSA, RectoSigmoid Aganglionosis; L-HSCR, Long Hirschsprung form; TCSA, Total Colonic Aganglionosis; RET, Rearranged During Transfection proto-oncogene. Post Severe, Severe HAEC following definitive surgery; Pre Severe, Severe HAEC before diagnosis; CAKUT, Congenital Anomaly of the Kidney and Urinary Tract; GUT, Congenital Intestinal Malformation; EYE, Eye abnormality*.

* *The asterisks in the new nomenclature recommendations indicate the stop codon. In particular it refers to the protein and the stop signal (https://varnomen.hgvs.org/)*.

## Results

### Overall Samples Distribution

During the study period, we admitted 41 consecutive HSCR patients. Due to the technical difficulties in frozen storage, only 31 of these patients provided adequate material to be sent to Bethesda (NIH, USA) for metagenomics. Of these 31 patients, only 20 provided both preoperative and postoperative specimen that passed internal quality controls. To summarize, out of 41 eligible HSCR patients, preoperative and postoperative stool samples were collected from 20 HSCR patients, 13 suffering from Rectosigmoid Aganglionosis (RSA), 1 L-HSCR and 6 Total Colonic Aganglionosis (TCSA). Median age at enrollment of these 20 patients was 16 months (ranging between 2 months and 13 years). A total of 40 stool samples (20 preoperative and 20 postoperative) were sequenced for 16S rDNA. In order to increase results reliability and to address HSCR forms with the highest risk of HAEC occurrences, we mostly focused on patients with TCSA that represented the core of our study (Table 1).

### Demographics of TCSA Patients

Six patients with TCSA were included. Male to female ratio was 2:1. Median age at enrollment was 15.5 months (range 9 to 26 months). *RET* mutations were detected in 7 out of 20 patients (35%), 4 of whom suffering from TCSA (67%). Three of these 4 nucleotide changes lead to truncating mutations and, interestingly, none was associated with HSCR cases complicated by HAEC manifestations. Two patients reported a previous history of HAEC (subject A and K), one (subject C) developed HAEC postoperatively well after postoperative timepoint (Table 1). *RET* mutations did not correlate either with protection or predisposition to HAEC (*p* = 0.4000). Four associated anomalies were detected in 3 patients. These included Congenital Anomalies of the Kidney and Urinary Tract (CAKUT) (*n* = 2), Gastrointestinal anomalies (*n* = 1), and Visual Impairment (*n* = 1) (Table 1).

### Microbiota Diversity and Composition Comparing TCSA to RSA (20 Patients, 40 Samples)

#### Diversity

**Figure 1:**
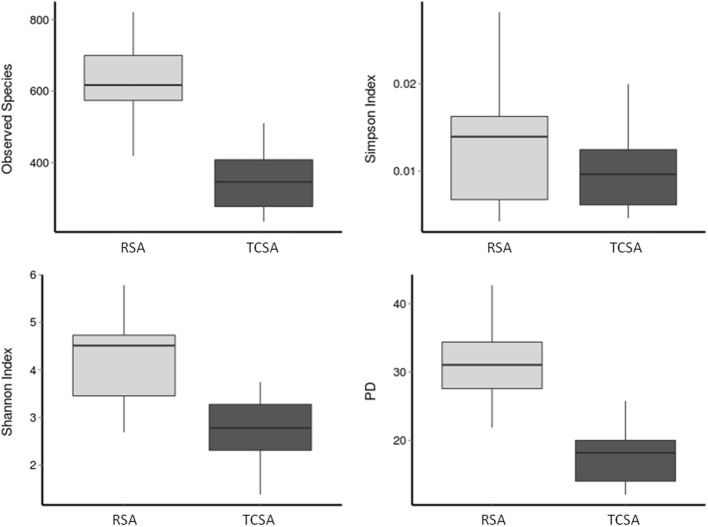
The diversity of gut microbial communities was assessed through four alpha diversity measures. Together, these results suggest that ileal stools belonging to TCSA carry lower microbiota diversity.

The diversity measures (Figure 1) were calculated based on the results derived by the overall assessment of specimen provided by each group (13 RSA and 6 TCSA, both timepoints). The diversity of gut microbial communities was assessed with alpha diversity measures. We calculated the number of observed species-level OTUs in each sample and found that the gut of RSA patients contained a higher number of species-level OTUs than the gut microbiota of TCSA patients (mean difference = 278 species; *p* < 0.001). Similarly, the Shannon index (35), which measures the richness and evenness of a community, was higher in RSA patients than TCSA patients (mean difference = 1.51; *p* < 0.01). Phylogenetic Diversity (PD) (39), which takes into account phylogeny, further confirmed our findings that RSA patients possessed greater microbial diversity compared to TCSA ones (mean difference = 13.42; *p* < 0.005). However, the Simpson Index (38) did not significantly differ between RSA and TCSA patients. The Simpson index measures diversity by calculating the evenness of OTUs in each community and penalizes communities dominated by a small number of OTUs (Figure 1).

#### Composition

**Figure 2:**
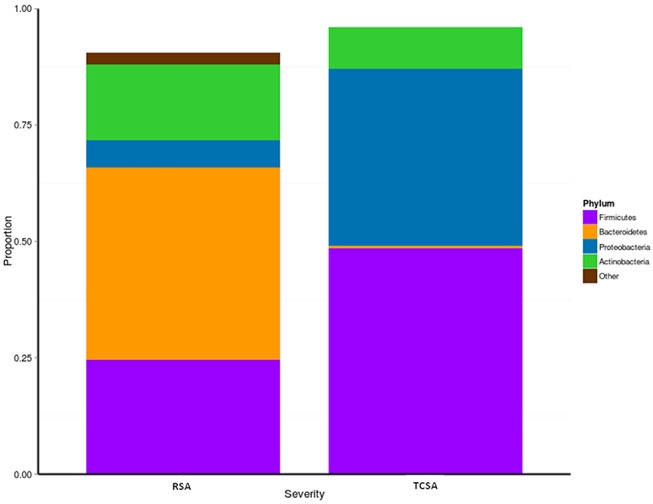
We assessed microbial composition by comparing the relative abundances of taxa at phylum level. When considering the communities at the phylum level, differences between RSA and TCSA microbiota were clearly evident. Bacteroidetes represented over 33% of all bacteria in RSA and where basically absent (<2%) in TCSA. Similarly, Proteobacteria accounted for nearly 40% of all bacteria in TCSA and for <5% in RSA. Conversely, Firmicutes and *Actinobacteria* were present both in RSA and TCSA without statistically significant differences.

We assessed microbial composition by comparing the relative abundances of taxa at the phylum and genus level. At the phylum level differences between RSA and TCSA microbiota were even more striking, specifically with regard to the presence of Bacteroidetes (Figure 2). Nearly 70% of RSA patients (69%) had communities composed of over 33% Bacteroidetes whereas all TCSA communities contained <2% Bacteroidete*s*, which was found to be a significant difference (mean difference = 40.83%; *p* < 0.05). A greater presence of Proteobacteria in TCSA microbiota compared to RSA microbiota was also observed (mean difference = 32.27%; *p* < 0.05). The remaining two classifiable phyla found in all of the samples, Firmicute*s* and Actinobacteria, did not significantly differ in relative abundance between the two groups.

When considering the communities at the genus level, we observed a prominent composition (>25%) of *Bacteroides* in the majority of the RSA patients' gut microbiota, whereas all of the TCSA patients contained <1% of *Bacteroides* in their gut microbiota. Similarly, we observed a significant increase of *Alistipes* (a genus in the same Phylum as *Bacteroides*, Bacteroidetes) in the gut microbiota of RSA patients when compared to the TCSA patients (mean difference = 1.76%; *p* < 0.05). Conversely, we found that members of the genus *Enterococcus* (a member of the Firmicutes Phylum) were more prevalent in the microbiota of TCSA patients (mean difference = 2.44%; *p* < 0.05).

### TCSA Patients (6 Patients, 12 Samples)

**Figure 3:**
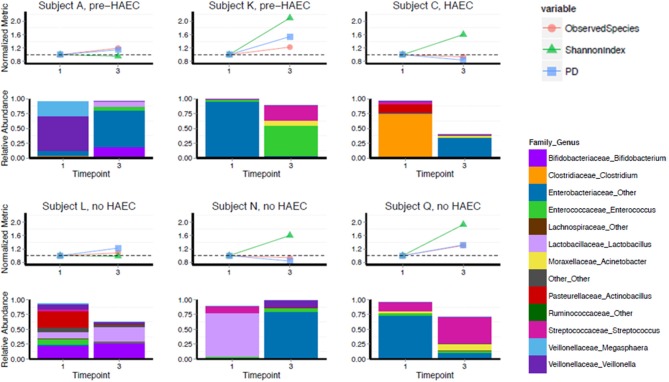
Diversity and composition in TCSA before and after surgery. X-axis depicts timepoints of sample collection: pre-operative (“1”) and post-operative (“3”). Subjects A, K, and C either developed HAEC postoperatively (pre-HAEC—HAEC status at stool sampling) or preoperatively (HAEC—HAEC status at stool sampling). Subjects L, N, and Q never developed. The top graph of each panel represents alpha diversity, normalized to values of sample collected pre-operatively, with each color line representing a different metric. The dotted line on each graph is set to 1.0. The bottom panel illustrates relative abundance of taxa at genus level.

Gut microbiota of 6 HSCR patients with TCSA was assessed before and after surgery (comparing timepoints) performed to restore bowel continuity and reverse the stoma (Figure 3). All preoperative sampling were obtained by the stoma. Three patients (Subjects L, N, and Q) never experienced episodes of HAEC. One patient (Subject C) experienced an HAEC episode preoperatively, while the two remaining patients experienced post-operative HAEC episodes (Subjects A and K).

We analyzed changes in alpha diversity between the two timepoints using three metrics: OTU richness (the number of observed OTUs in a sample), Shannon Diversity (a measure of OTU richness and evenness), and PD whole tree (a metric that takes phylogeny into account). Four of six subjects increased in Shannon diversity post-operatively. The two subjects that did not increase in Shannon diversity (Subjects A and L) were stable by this metric as well as number of OTUs and PD whole tree. For PD whole tree and OTU richness, most patients also trended toward an increase, though two subjects remained stable or slightly decreased by these metrics (Subject C and Subject N).

Though our sample size was not large enough to adequately power statistical comparisons, we noted that composition of the gut microbiota was highly variable, both pre- and post-operatively, regarding genus level taxonomy. Pre-operatively Subject A was predominantly colonized with *Veillonella*, Subjects K and Q were predominantly colonized by *Enterobacteriaceae* family, Subject C was predominantly colonized with *Clostridium*, and Subject N was predominantly colonized with *Lactobacillus* genus. Subject L was not dominantly colonized by a single genus level taxa and contained a more diverse mixture. For all subjects, there was a drastic shift in composition post-operatively, though we did not detect any specific trend in this regard. At a phylum level, Proteobacteria showed a striking predominance over other phyla either preoperatively or postoperatively but without relationship with HAEC occurrences or HAEC status. In fact, HEAC status, as well as genetic background and phenotype did not show clear correlations with gut microbiota both pre- and post-operatively (Spearman correlation, *p* = 0.40; see Table 1, Figure 3).

## Discussion

Our study was aimed at addressing the effect of ultralong aganglionosis (TCSA) and its multiple implications, namely genetic background (higher prevalence of *RET* mutations), motility issues (higher prevalence of obstructive symptoms), and HAEC occurrences [higher frequency of HAEC episodes (12, 21, 22)] on gut microbiota composition. In fact, though it is still unknown if those variables are independent one from the others, RSA and TCSA must be considered separately when addressing HSCR patients.

Only a few published papers compared ileal and colonic fecal microbiota in children with HSCR-associated enterocolitis (HAEC). Available studies mostly refer to newborns or ex-preterm with a history of necrotizing enterocolitis or to children with inflammatory bowel diseases (41–45). The only study comparing microbiota of ileal and colonic stools in healthy subjects, is that by Zoetendal et al. in 2012 (46) but the authors included only adult subjects. Even so, our study confirmed that ileal samples have a lower microbiota diversity when compared to colonic ones, regardless of the presence of a stoma or not, in accordance to work reported by Zoetendal et al. (46). Recently, Barret et al. compared two preterm babies with different stomas (ileal and colonic) and followed them up to 7 months of age (41). They reported that Bifidobacteria and *Enterobacteriaceae* dominate at a genus level in the ileal stools samples (41). Our results are consistent with these findings with similar preponderances at a genus level. Of note, statistical analysis failed to show significant differences in terms of diversity and composition with relation to HAEC occurrences in TCSA patients, thus confirming the heterogeneity of gut microbiota and the difficulty in finding a clear and univocal marker for HAEC predisposition.

Nonetheless, the lower diversity and variable composition of microbiota throughout time points confirms that either with a stoma or not, patients with TCSA have different gut microbiota compared to those with RSA. This aspect could be easily expected basing on previous reports concerning microbiota diversity and composition in different bowel sites (41–46). Even so, as TCSA patients are well known to be susceptible to HAEC (12, 21, 22, 47), we speculate that either reduced diversity in TCSA patients can be merely related to a different sampling site or that this aspect has a pathogenetic role in terms of HAEC predisposition/facilitation. Alternatively, the well-known dysmotility and fecal stasis observed in TCSA patients could lead to bacterial overgrowth that can interfere with homeostasis and bacterial dynamics even more. In case of bacterial overgrowth, a potentially harmful microbial species can outcompete commensals very rapidly and lead to HAEC as a result of systemic reaction to this dysbiosis. This argument is supported by the factthat we could not observe a specific genus or phylum significantly associated with HAEC occurrence within TCSA patients. Nonetheless, our series of patients with TCSA proved to have a significantly higher abundance of Proteobacteria when compared to RSA ones. In particular, 5 out of 12 samples belonging to TCSA patients were more than 80% composed by Proteobacteria. Conversely, Bacteroidetes were basically absent. The total loss of the colon undermines the existence of a specific organ, composed both of mammalian cells and microbial cells. The colon microbiota provides the system with a fundamental stabilizing function, of crucial importance for the robustness of the whole microbial and immune system. The colonic diversity and richness creates a resilient, reliable, and robust microbial community that can easily cope with potential insults, including the colonization from environmental and food microbiota. In this context, the absence of Bacteroidetes affects the production of short chain fatty acids (SCFAs) which are fundamental for intestinal homeostasis. Our result is thus in agreement with a recent report that showed how fecal samples from HAEC children showed a 4-fold decline in total SCFA concentration vs. non-HAEC HSCR patients (48). In particular, the authors found reduced acetate and increased butyrate in HAEC children, with 10 of 12 butyrate-producing genera as well as 3 of 4 acetate-producing genera demonstrated multi-fold expansion. Yet, we cannot demonstrate whether Proteobacteria preponderance and Bacteroidetes deficiency are linked to sampling site (ileal vs. colonic) or to HAEC predisposition, as recently suggested by Li et al. (15). Noteworthy, sampling site of their HAEC patients belonged more frequently to ileum or right colon, thus introducing a significant bias in the interpretation of their results (15). In TCSA, the ecological resilience of the microbiota to resist an insult is deeply undermined. Thus, TCSA patients have a fragile ileal microbial community, which may be extremely sensitive to dysbiosis that is otherwise harmless in a normal system. This is evident not only by the recurrence of HAEC, but also the variations of microbial compositions between individual patients.

This result is shown not only by the recurrence of HAEC but also from the extreme interindividual variability of the microbial composition. In absence of the colon TCSA patients have a highly variable microbiota, lacking fundamental species associated to heath in normal individuals, such as Bacterioidetes. The abundance of Proteobacteria also reflects the invasion from environmental communities, that are free to thrive in an environment that would be normally precluded to them by the presence of Bacterioidetes. Thus, HAEC should be seen not as a response to a pathogen, but as a response to a community that normally should not be present in the ileum, or in the colon, eliciting deleterious consequences. This explanation of our results is further supported by the most striking finding of a recent study on HAEC in a mouse model of HSCR that suggested how *Akkermansia*, a microorganism normally seen as protective, may contribute to development of enterocolitis while Bacteroidetes may be protective. Less abundant genera that were reduced in HAEC were *Dysgonomonas* and *Clostridium XIVa*, which may play a protective role (16). Taken as a whole our findings suggest that the use of therapies targeted at *Clostridium difficile*, without sufficient confirmation for *Clostridium difficile* overgrowth, might be detrimental in patients with HAEC following total colon resection. Even so, it is still possible that certain composition of the intestinal microbiota, as that reported by Li et al. or in our study, can predispose to HAEC in case of a susceptible genetic and immunologic background well known to be significantly more frequent in TCSA (22). In agreement with previous results (49) 65% of TCSA and 20% of RSA patients in our study have *RET* mutations. As recently published by our group (50), these mutations could determine abnormal expression of RET-dependent and independent pro-inflammatory programs that might predispose to HAEC occurrence. *RET* sequencing in our study could not be correlated to the incidence of HAEC mostly due to the limited number of HAEC-TCSA in our series of patients. We speculate that the interaction of a less diverse and compositionally peculiar gut microbiota (preponderance of Proteobacteria over Bacteroidetes) in patients with an imbalanced RET-dependent and independent immunity (regardless of the loss-of-function effect of *RET* mutations) could facilitate HAEC onset and/or predisposition. In this perspective, the defects observed in HSCR are not restricted to the aganglionic segment but extend to the mucosal immune system within and beyond the gastrointestinal tract, including the microbiota composition (50).

Although our study underlined the potential of metagenomics and improves the understanding of the relationship between microbiota, host, and immune system in patients with HSCR, it suffered important limitations. First of all, although all patients were sampled relatively far away from antibiotic therapy, we cannot exclude long term effects of antibiotic treatment. The microbiota composition proved to be patient-specific and likely depend on patients' personal history, as previously reported by Barrett et al. and Zoetendal et al. (41, 46). In particular, genera and phyla were heterogeneous in patients from our series and there was no specific and reproducible common pattern according to length of aganglionosis, genotype, phenotype, and HAEC status. This was evident when we addressed stools composition of TCSA patients with and without HAEC whose relative abundance of bacterial taxa at each time point were basically not comparable. In context of bacterial diversity and community dynamics, we could observe some of the most intriguing potentials of metagenomics in HSCR, all pointing to a lack of robustness in the gut microbiota. We argue this is due to the loss of the organ principally responsible for maintaining the reservoir of those microbes providing the buffer effect, with a loss of biodiversity corresponding to a loss of resilience. On this specific regard, it appears extremely important to achieve a heathy status by reconstituting this microbial buffer, in order to re-establish a minimal resilience and homeostasis. On the ground of these considerations, strategically designed fecal transplant defined by composition and the abundance enjoy exciting potential to address this issue in TCSA patients.

To conclude, our study confirmed the enormous potential of metagenomics in HSCR but underlined the importance of identifying the proper subset of patients for this powerful methodology. Based on the prevalence of HAEC, patients with TCSA represent the ideal subgroup to study HAEC susceptibility. A longitudinal long-term study on high-risk patients will presumably provide information that could be better compared, analyzed, and possibly applied or transferred to the general HSCR population. At present, we can only speculate that higher biodiversity could play a role in maintaining gut homeostasis and that its disruption could facilitate HAEC development.

## Ethics Statement

Institutional Ethical committee approval was obtained by the Review Board of Giannina Gaslini Institute on November 2009 as part of a wider research project on HSCR.

## Author Contributions

AP formulated the idea, designed the study, and wrote and reviewed the manuscript. CB-M performed the DNA extraction and sequencing and analysis. MM coordinated HSCR patients sample acquisition, biobanking, and shipping HSCR patients. VR, IC, SA, and MF sampled stools from HSCR patients and coordinated patients' phenotype and genotype assessment. KH revised the manuscript drafts and supported the lab work. GM revised the manuscript draft and supervised patients data acquisition. FL performed the molecular genetics studies. DL, WP, and DM conceived the study and drafted the manuscript. CD and MD analyzed the microbiome data. DC reviewed the partners contributions, drafted the manuscript, analyzed the microbiome data, and inspired the study.

### Conflict of Interest Statement

The authors declare that the research was conducted in the absence of any commercial or financial relationships that could be construed as a potential conflict of interest.

## Supplementary Material

Raw data are available at the following link: https://drive.google.com/drive/folders/1MinrLjrlnuufdHRo7aKm1XuvOwNJvR6W.
